# Highly Specific Polyphenolic Colloids as Alternatives to Antimicrobials in Livestock Production

**DOI:** 10.3390/ijms25179363

**Published:** 2024-08-29

**Authors:** Andrea Laconi, Alessandro Cecconello, Simone Molinari, Graziano Rilievo, Aura Cencini, Federica Tonolo, Antonie Krystofova, Hardik Nilesh Majethia, Roberta Tolosi, Eliana Schiavon, Carlo Nicoletto, Alessandra Piccirillo, Fabio Vianello, Massimiliano Magro

**Affiliations:** 1 Department of Comparative Biomedicine and Food Science, University of Padua, Viale dell’Università 16, 35020 Legnaro, Italy; andrea.laconi@unipd.it (A.L.); alessandro.cecconello@unipd.it (A.C.); graziano.rilievo@studenti.unipd.it (G.R.); aura.cencini@studenti.unipd.it (A.C.); federica.tonolo@unipd.it (F.T.); antoniekrystofova@gmail.com (A.K.); hardiknilesh.majethia@studenti.unipd.it (H.N.M.); roberta.tolosi@unipd.it (R.T.); alessandra.piccirillo@unipd.it (A.P.); fabio.vianello@unipd.it (F.V.); 2 Department of Geosciences and CIRCe Centre, University of Padua, Via G. Gradenigo 6, 35129 Padua, Italy; simone.molinari@unipd.it; 3 Istituto Zooprofilattico Sperimentale delle Venezie, Viale dell’Università 10, 35020 Legnaro, Italy; eschiavon@izsvenezie.it; 4 Department of Agronomy, Food, Natural Resources, Animals and Environment, University of Padua, Viale dell’Università 16, 35020 Legnaro, Italy; carlo.nicoletto@unipd.it

**Keywords:** nanoparticles, antimicrobials, wood industry by-products, AMR

## Abstract

The dispersion of antibiotics in livestock farming represents a health concern worldwide, contributing to the spread of antimicrobial-resistant bacteria through animals, the environment, and humans. Phenolic compounds could be alternatives to antibiotics, once drawbacks such as their low water solubility, bioavailability, and reduced stability are overcome. Although nano- or micro-sized formulations could counter these shortcomings, they do not represent cost-effective options. In this study, three phenolic compounds, obtained from wood-processing manufacturers, were characterized, revealing suitable features such as their antioxidant activity, size, and chemical and colloidal stability for in-field applications. The minimum inhibitory concentration (MIC) of these colloidal suspensions was measured against six bacterial strains isolated from livestock. These particles showed different inhibition behaviors: Colloidal chestnut was effective against one of the most threatening antibiotic-resistant pathogens, i.e., *S. aureus*, but ineffective toward *E. coli*. Instead, colloidal pine showed a weak effect on *S. aureus* but specificity toward *E. coli*. The present proof-of-concept points at colloidal polyphenols as valuable alternatives for antimicrobial substitutes in the livestock context.

## 1. Introduction

Livestock farming is an integral part of the food industry, providing protein sources such as meat, milk, and eggs, which are further processed into products and by-products destined for human consumption [1]. Bacterial infections generate substantial detrimental effects that not only involve animal health but also have significant implications for welfare, productivity, and the industrial chain. The use of antibiotics played a crucial role in preventing and treating bacterial infections, promoting animal health, and ensuring optimal productivity. However, their overuse and misuse has contributed to the emergence and spread of antibiotic-resistant bacteria, such as *Escherichia coli* resistant to third- and fourth-generation cephalosporines or methicillin-resistant *Staphylococcus aureus* (i.e., MRSA) [2]. Zoonotic diseases, which are transmitted from animals to humans, pose significant concerns in livestock production. The consumption of contaminated food, such as meat or dairy products obtained from animals hosting resistant bacteria, can contribute to their transmission to humans, posing a threat to public health [3]. Infections caused by resistant bacteria are more difficult to treat, leading to prolonged illness, increased healthcare costs, and higher mortality rates [4]. Addressing antimicrobial resistance requires promoting the responsible use of antimicrobial drugs, implementing surveillance programs, and exploring alternative approaches [5]. Polyphenols are widely recognized for their antioxidant and anti-inflammatory effects, which improve human health and contribute to disease prevention [6,7]. The antibacterial activity of polyphenols is believed to be related to the expression of specific genes in bacteria, leading to alterations in bacterial metabolism and virulence. On these bases, the potential for naturally occurring polyphenols to become alternatives to antimicrobials has been explored [8]. These molecules downregulate the expression of genes involved in pathogenicity and upregulate the expression of genes related to stress response and antimicrobial resistance [9]. Furthermore, polyphenolic compounds have shown potential as anti-biofilm agents [10] and antimicrobial effects against foodborne bacteria, including Gram-positive and Gram-negative bacteria, and fungi [11].

Even though polyphenols have demonstrated an outstanding antibacterial potential, most of them present poor water solubility, short pharmacokinetics half-lives, and low bioavailability [12]. Furthermore, environmental factors such as acidity, temperature variations, and enzymes compromise the stability of these substances, thereby further limiting their therapeutic activity. Nano-formulations have reduced drastically the existing limitations to polyphenol use. For instance, they have resulted in enhanced bioavailability and controlled drug release, improving their suitability for human biomedical use and livestock production applications [13]. Moreover, the denser particulate acts as a protective shield for polyphenols, preventing their degradation, enhancing their tissue absorption, and minimizing their potential toxicity in animals and humans [14]. Examples of existing nano-formulations that have been previously studied to improve polyphenolic agents include polymeric nanoparticles, liposomes, hydrogels, microspheres, transferosomes, solid lipid nanoparticles, metal nanoparticles, drug–polymer conjugates, and nanostructured lipid carriers [14]. However, the translation of this kind of approach from human healthcare to the large-scale production of livestock food additives is far from being a trivial and easy-to-solve matter. When investigating their antimicrobial activities and the associated biological mechanisms, phenolic compounds have been commonly studied as chemicals, focusing on their molecular structures and reactivity [15]. Their direct action on the microbial cell wall has been attributed to epigallocatechin, while their inhibition of bacterial gene expression has been ascribed to catechin and their anti-biofilm activity to rosmarinic acid. Some of these mechanisms are not specific (oxidative stress), while others are specific [14,15].

This view does not consider the fact that some natural phenolic compound sources, such as wood, when subjected to chemical treatments and strong mechanical stress, typical of large-scale manufacturing plants, can generate micro- and even nano-sized materials [16]. In other words, inexpensive polyphenolic micro- and nanoparticles are already available as by-products of industrial production, for instance, after the processing of natural materials such as wood, offering cost-effective antibiotic competitors for livestock infection management. It is worth mentioning that wood-derived materials have been tested as antimicrobial agents, but their chemical–physical features such as their hydrodynamic size and zeta potentials have usually been neglected during the interpretation of their action mechanisms [17]. These large-scale-produced phenolic materials may display adequate chemical and colloidal stability, which is a crucial pre-requisite for their in vivo application. Furthermore, their characterization could be helpful for understanding their antimicrobial activity. Based on this rationale, three compounds obtained during the industrial processing of wood were first characterized, revealing optimal chemical–physical and colloidal characteristics. These colloidal materials were named depending on their respective source, as follows: colloidal chestnut A and B (CCA and CCB), derived from chestnut trees from two distinct Italian regions, and colloidal pine (CP), from pine biomass. They were tested on six bacterial strains isolated from cattle, swine, and poultry (i.e., *Pasteurella multocida*, *Staphylococcus aureus*, *Streptococcus suis*, *Mannhemia haemolitica*, *Escherichia coli*, and *Salmonella enterica* serovar Typhimurium).

Colloidal chestnut A (i.e., CCA) and colloidal pine (i.e., CP) displayed the most divergent behaviors, with opposite antioxidant powers and highly different specificities regarding their highest bacteriostatic performances. Although the observed selectivity is far from being easily explained, it points at the need of conceiving tailored phenolic combinations as possible alternatives to antimicrobials in livestock. Moreover, the colloidal perspective presented here paves the way for further studies, overcoming the limited vision of phenolic compounds as merely having molecular-related activities.

## 2. Results

### 2.1. Morphological and Hydrodynamic Characterization

**Figure 1:**
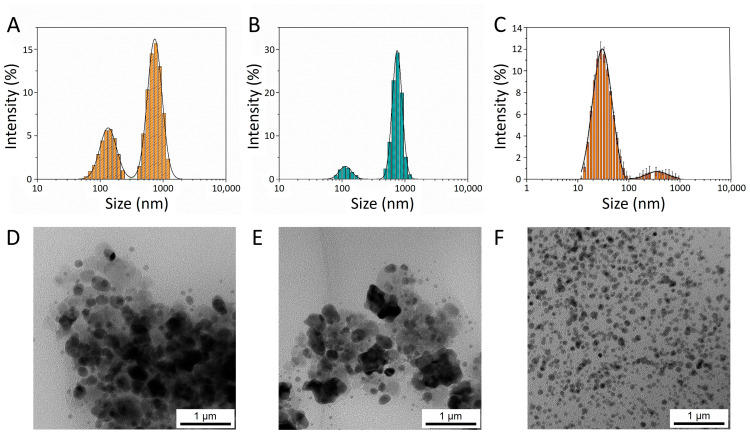
Hydrodynamic and morphological characterization of the colloidal phenols. DLS hydrodynamic radii of (**A**) CCA, (**B**) CCB, and (**C**) CP. TEM micrographs of (**D**) CCA, (**E**) CCB, and (**F**) CP.

The hydrodynamic radii and zeta potential (ζ) values of the carbonaceous materials were characterized by dynamic light scattering (DLS), as shown in Figure 1A–C. According to the DLS characterization, both chestnut polyphenols presented two distinct colloidal populations in the submicron and nano-sized regions. The estimated hydrodynamic sizes of colloidal chestnut A (CCA) were 147 ± 2 nm and 793 ± 3 nm, displaying an average zeta potential (ζ) of −14 ± 1 mV (conductivity 0.098 mS cm^−1^ in water at 22 °C), whereas the colloidal chestnut B (CCB)’s sizes were 120 ± 2 nm and 779 ± 1 nm, with an average ζ value of −14 ± 1 mV (conductivity 0.079 mS cm^−1^ in water at 22 °C). Interestingly, colloidal pine (CP) was characterized by a minor contribution from submicron particles and an abundance of nano-sized materials with dimensions in the region from 10 to 100 nm, overall displaying an average hydrodynamic radius of 280 ± 60 nm and a quite remarkable average ζ value of −23.8 ± 0.4 mV (conductivity 0.091 mS cm^−1^ in water at 22 °C). Indeed, the DLS observations were corroborated by TEM micrographs, shown in Figure 1D–F, illustrating that CCA and CCB were characterized by the relevant presence of submicron materials, while CP was predominantly represented by poli-dispersed and very small spherical nanoparticles.

Although possessing a contained ζ in the 10–30 mV interval and, therefore, being classifiable (either positively or negatively) in the range from moderately stable to stable [18], these suspensions presented no sign of precipitation during a six-months monitoring period and at concentrations in an aqueous milieu equal to 540, 340, and 50 g L^−1^ for CCA, CCB, and CP, respectively. Thus, although phenolic compounds are commonly insoluble in water, these particulate materials produced rather stable colloidal suspensions, suitable for application purposes and characterized by a significant fraction of nano-sized particles.

### 2.2. Chemical–Physical Characterization

**Figure 2:**
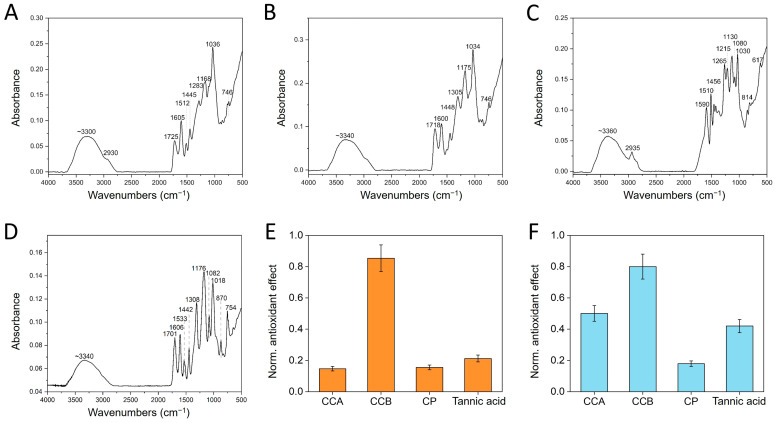
Chemical characterization of the phenol dried powders. FTIR profile of (**A**) CCA, (**B**) CCB, (**C**) CP, and (**D**) commercial tannic acid, used as a control. Comparison of the antioxidant powers through (**E**) FRAP and (**F**) Folin–Ciocalteâu assays.

In order to explain the colloidal nature of the investigated compounds, they can be assumed to consist of supramolecular structures in the nano- and micro-size range [19], in which phenolic constituents are held together by a number of non-covalent bonds, such as aromatic stacking, hydrogen bonds, and electrostatic interactions. In this view, Fourier transform infrared spectroscopy (FTIR) was used to characterize these self-assembled molecular aggregates from a vibrational standpoint, and the characteristic absorption wavelengths are reported in Table 1. All the wood-derived powders share very similar FTIR profiles, shown in Figure 2A–D, comparable to that of commercial tannic acid (TA), confirming an analogous chemical identity.

In order to provide a general description of this FTIR fingerprint, the overall vibrational contributions can be briefly summarized as follows: (1) a broad absorption between 3300 and 3360 cm^−1^ ascribable to the stretching vibrations of the hydroxyl groups -OH [20,21]; (2) a peak around 2930 cm^−1^ accompanied by a shoulder around 2850 cm^−1^, clearly distinguishable only in Figure 1D and attributable to the symmetric stretching vibrations and antisymmetric -CH- of the CH_2_ and CH_3_ groups [21]; (3) a strong band associated with the carbonyl group C=O stretching in the region from 1730 to 1705 cm^−1^ [21,22,23,24]; (4) the signals in the 1605–1500 cm^−1^ range can be assigned to the deformation vibrations of the carbon–carbon bonds of the aromatic backbone [21]; (5) a peak in the 1440–1460 cm^−1^ interval, due to the stretching vibrations of C-C aromatic groups; and (6) the characteristics bands of the C-O stretching vibration falling at 1100–1300 cm^−1^, with very pronounced peaks around 1100 cm^−1^ [21,24,25] (Figure 2A–D). A more punctual spectral attribution is listed in Table 1; in general, these results are similar to those reported in the literature for polyphenolic compounds such as tannic acid [22,26].

Although the spectra were comparable in terms of vibrational contributions, the wood-derived materials reported an intensity decrease in many peaks located in the range between 1750 and 750 cm^−1^ compared to tannic acid (Figure S2). In particular, these peaks were as sharp and strong as the ones observed in the control. This can be related to a higher degree of rigidity due the aggregated character of the molecules involved in the supramolecular assemblies, namely the documented nano- and microparticles (see above).

In Figure 2E,F, it is possible to appreciate the antioxidant power of the colloidal phenols in comparison to commercial tannic acid according to the FRAP and Folin–Ciocalteâu assays, respectively, highlighting CCB as the most effective material within the examined group. Since the morphological and hydrodynamic characterization of the materials was interpreted as the result of the supramolecular clustering of polyphenols, kept together by non-covalent interactions, preliminary chemical stability tests were carried out by incubating the dried powders in different conditions, and the effect of the chemical environment on the material was monitored thorough DLS analysis. The incubation in 96% ethanol led to the slow dissolution of the powders, confirming the hydrophobic intermolecular interactions. Noteworthily, as the particulate disappeared, the liquid turned into a progressively darker suspension. Upon exposure to a strong alkaline milieu, such as 3 M NaOH, the powders instantaneously dissolved and turned into a black material. Meanwhile, the powders were not susceptible to acidic conditions up to 3 M HCl. This can be explained by assuming that, under acidic conditions, molecular hydroxyl groups are non-ionized. Differently, when the pH was shifted to alkaline values, the materials dissolved as a consequence of the electrostatic repulsion that took place at a molecular level between single polyphenols, as well as because of the increase in their hydrophilicity, being negatively charged due to hydroxyl group deprotonation [27].

**Figure 3:**
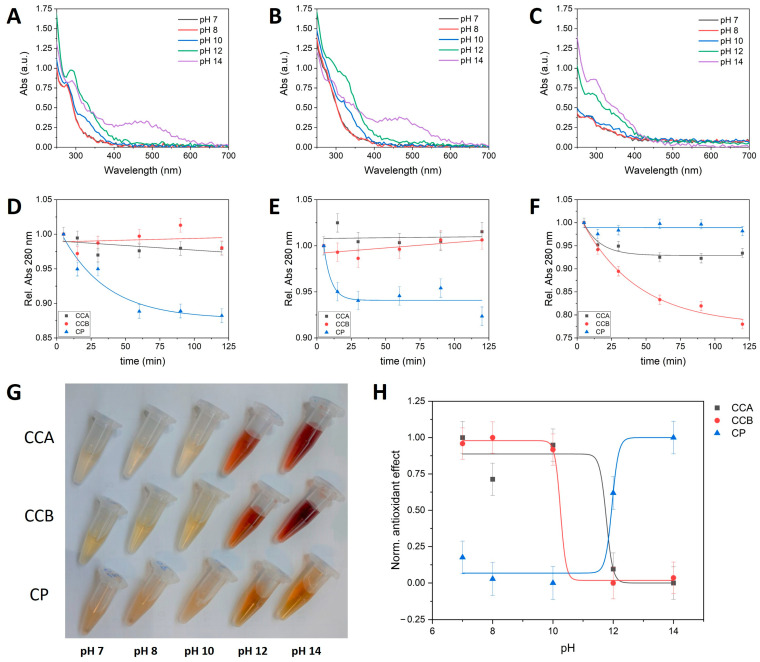
Study of the disaggregation and oxidation behaviors of CCA, CCB, and CP. The effect of increasing pH values documented by UV-vis spectroscopy for (**A**) CCA, (**B**) CCB, and (**C**) CP. Comparison of the evolution of the aromatic phenol ring’s UV-vis peak at 280 nm as a function of time at (**D**) pH 7, (**E**) pH 8, and (**F**) pH 12. (**G**) Comparison of color variation as a function of the pH. (**H**) Comparison of antioxidant powder as a function of the pH.

Overall, when the particulate form was degraded into its molecular constituents, as proven by the disappearance of the aforementioned particulate by DLS analysis, the former colloidal polyphenol was rapidly oxidized into a black material showing no antioxidant power according to the Folin–Ciocalteâu assay. On these bases, with the aim of marking possible differences among the three colloidal compounds, a more in-depth analysis of the disaggregation and oxidation behaviors was carried only in alkaline conditions. Hence, the pH influence was explored in the 7–14 range and as a function of time using DLS, UV-vis, and measuring the antioxidant through the Folin–Ciocalteâu method. In Figure 3A,B, it is possible to appreciate the evolution of the CCA’s and CCB’s UV-vis profiles with the increase in the pH and, in particular, the intensity of a band around 280 nm, attributable to the phenol aromatic rings, progressively diminishing as the alkalinity increases, almost fully disappearing at a pH of 14. In its place, the appearance of a new chromophore characterized by a broad band around 450 nm is registered and ascribed to the occurrence of oxidation phenomena. Indeed, the emergence of a very similar spectral feature was reported for analogous chestnut materials upon the action of a strong oxidant [28]. The DLS measurements confirmed that the samples, in these pH conditions, were below the limit of detection of the instrument, prompting that the colloidal materials were disaggregated into their molecular components and likely became susceptible to oxidation. Conversely, CP showed an increasingly more pronounced aromatic band at 280 nm in the examined pH range, accompanied by the appearance of a new chromophore signal already visible at a pH of 12, in the form of a broad shoulder centered around 350 nm and ascribable to oxidation processes (Figure 3C) [29]. This can possibly be explained by considering the simultaneous occurrence of disaggregation and oxidation. However, differently from chestnut, a prevalence of the disaggregation phenomenon could be evinced observing the progress of the pine UV-vis profile, along with the pH enhancement. Disaggregation was again witnessed through the disappearance of colloidal particles, as substantiated by the DLS analysis.

From the comparison of the kinetic behavior, monitored following the aromatic UV-vis fingerprint at 280 nm, it can be noted that the feature is influenced by the pH in the case of CCA and CCB, but it is almost unaffected in the case of CP (Figure 3D–F). The aforementioned emergence of new chromophores, witnessing the occurrence of oxidative processes, can be immediately appreciated by observing the drastic color change in the chestnut and pine contents in the experimental tubes, turned red and yellow, respectively, fully in harmony with the band wavelengths documented by UV-vis characterization (Figure 3G). Finally, in good agreement with the optical analysis, the antioxidant power of the chestnut compounds registered a decrease above a pH of 10. In particular, CCB, the compound showing a more pronounced pH susceptibility according to the decrease in the 280 nm UV-vis signal, was also the one registering a decrease in its antioxidant power already at a pH of 10. CCA showed a more contained decrease in the phenol aromatic ring’s UV-vis feature, and its antioxidant power was well-conserved until a pH of 12. Instead, pine showed an increase at approximatively the same pH, confirming that the oxidation phenomenon was apparently more relevant for chestnut than pine. In this range of pH, the antioxidant power against the pH roughly followed a sigmoidal trend for all the examined compounds (Figure 3H). Thus, although it has been highlighted that it is the physical structure of these compounds that preserves the molecular cargos from degradation, it is unlikely that an actual antimicrobial effect could be exerted without the disaggregation of these supramolecular clusters. Hence, the different disaggregation–oxidation trends could be related to distinct antimicrobial activities (see below). Moreover, all these compounds gave rise to long-term stable water suspensions, combining the aforementioned chemical stability in normal environmental conditions, with colloidal properties which are suitable for in vivo and in-field applications.

### 2.3. Minimum Inhibitory Concentration (MIC) of Phenolic Compounds on Selected Bacteria

**Figure 4:**
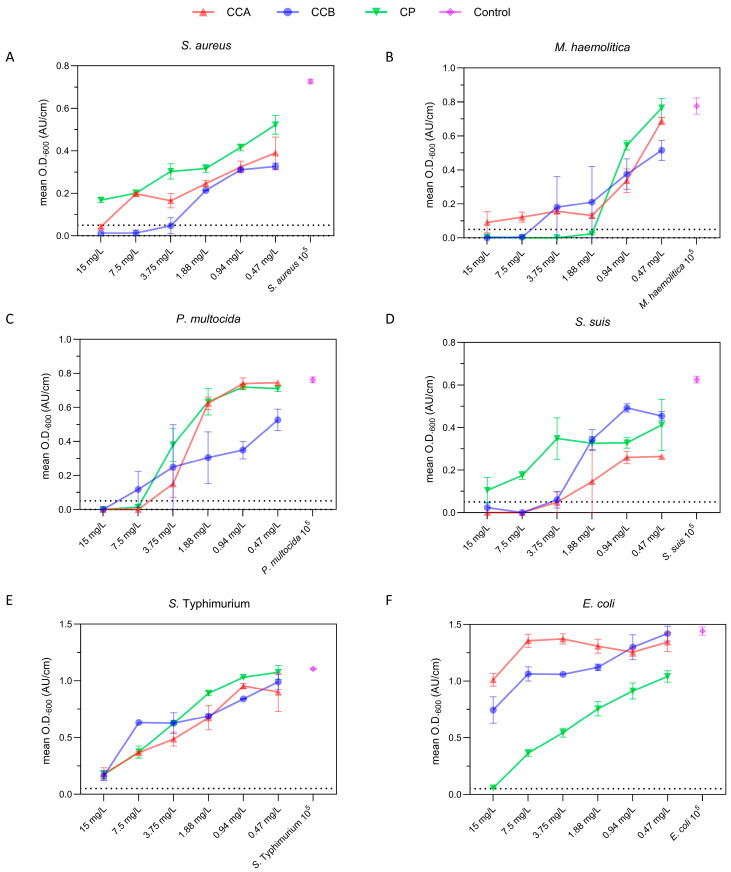
MIC determination for CCA (blue), CCB (red), and CP (green) against *S. aureus* (**A**), *M. haemolytica* (**B**), *P. multocida* (**C**), *S. suis* (**D**), *S.* Typhimurium (**E**), and *E. coli* (**F**). Each point in a logarithmic horizontal axis of colloid concentrations represents the mean O.D. of six independent replicates, with the vertical whiskers indicating the standard deviation (SD). The horizontal dashed lines represent the MIC threshold set at a 0.05 increase in O.D. Bacterial strains inoculated without any compound were used as positive controls and are shown in purple.

The minimum inhibitory concentration (MIC) is a widely accredited parameter for conveying the effectiveness of antimicrobial agents, consisting in the lowest concentration of a chemical, usually a drug, able to prevent the growth of bacteria or fungi in vitro [30,31]. The inhibitory effect of phenolic particulates was assessed against selected bacterial strains, isolated from livestock, i.e., *Pasteurella multocida*, *Staphylococcus aureus*, *Streptococcus suis*, *Manneimia haemolitica*, *Escherichia coli*, and *Salmonella enterica* serovar Typhimurium (Table S1). The MIC of each compound was estimated using a microtiter assay by measuring the variation in the optical density at 600 nm (O.D._600_) by UV-vis spectrometry, after 24 h of incubation at 37 °C. In Figure 4, for each bacterial strain, the mean absorbance of six independent replicates is plotted against the polyphenol concentration in the range between 0.47 and 15.00 mg L^−1^.

Overall, the three materials displayed a concentration-dependent effect in the whole examined range, and, most importantly, the growth-inhibitory effect was significantly variable as a function of the specific pathogen. In order to better discriminate the variability in the effect exerted by the colloidal compounds and the group of investigated bacteria, the differences in the mean O.D._600_ were calculated (Table 2).

## 3. Discussion

A number of reported examples substantiated that an essential condition to obtain antibacterial efficacy is to endow a particle with a positive surface charge, as the latter allows for more efficient interactions with the bacterial cell wall [32]. As an example, surface-modified silver nanoparticles can attach to the bacterial wall and subsequently penetrate it, causing structural changes and leading to cell death [33]. Indeed, as a consequence of the ionized substituents exposed to the extracellular environment, the surfaces of bacterial cell possess net-negative electrostatic charges [34]. This facet clearly contrasted with our experimental data: although the three phenolic materials which are the object of the current study show suitable colloidal properties for in-field applications, they have a negative zeta potential value in common. Thus, charge repulsion is likely to occur at the abiotic–biotic interface, hampering the contact between the colloidal phenols and the target microorganisms. That said, notable exceptions to the aforementioned paradigm are available in the literature, and negatively charged materials with antibacterial activity can be found [35]. While the phenomenon observed in this study is far from being easily interpreted, one could imagine that the colloids may disaggregate into their reactive molecular constituents in the proximity of the microorganisms. Although disaggregation–oxidation processes could be driven by factors other than the pH in a real biological situation, alkalinity was a simple strategy for unveiling some distinctive traits in the investigated colloids. As emerged from the current chemical–physical characterization, these materials displayed completely different disaggregation–oxidation behaviors. Interestingly, CCB displayed the best overall antimicrobial performance, and it showed the highest antioxidant power, according to both the FRAP and Folin–Ciocalteâu assays, despite being the most unstable among the investigated compounds. However, this reactivity-focused interpretation is not sufficient to explain all the examined cases. *E. coli* was not significantly susceptible to CCB, but it was strongly inhibited by the chemically stable CP. Indeed, the correlation of the antimicrobial activity of this kind of colloidal materials and their chemical–physical characteristics deserves several dedicated studies.

Overall, an inhibitory effect was observed in the examined range of colloid concentrations, and it is worth noting that this trend significantly differed depending on the specific colloid–bacterium pair.

In Figure 4A, the growth of *S. aureus* is inhibited in the presence of 7.50 mg L^−1^ and 15.00 mg L^−1^ of CCB and CCA, respectively, revealing a remarkable efficacy toward one of the most persistent and detrimental microorganisms worldwide. Indeed, this bacterium is known to be resistant to multiple antibiotics, making its eradication a challenging task [36,37]. On the other hand, the third compound, CP, was only able to exert a weak effect on this bacterium at the tested concentrations. In turn, *M. haemolytica* displayed susceptibility to CP at as little as 1.88 mg L^−1^, while only partial inhibition was observed for CCA in the whole examined range of concentrations. Meanwhile, the MIC for CCB against *M. haemolytica* was 7.50 mg L^−1^, which was a significantly higher value compared to that of CP, prompting a higher specificity of the pine-derived material for this bacterial species. *M. haemolytica* is a major livestock pathogen, which can harbor multiple resistant determinants and cause severe respiratory diseases in farm animals [37,38], resulting in weight gain reduction, decreased feed efficiency, and overall impaired productivity in livestock [38]. Inhibition against *P. multocida* was obtained by all compounds, with concentrations ranging from 15.00 mg L^−1^ (CP) to 7.50 mg L^−1^ (CCB and CCA, Figure 4C). Taken together, these results suggest that CP exhibited a selective antibacterial activity against the family of Pasteurellaceae (*P. multocida* and *M. haemolytica*). Considering the high level of antimicrobial resistance commonly found in these two bacterial species, these findings are of great interest for both animal and human health. Indeed, *P. multocida* not only causes respiratory diseases in livestock animals, but it can also be transmitted to humans [39,40], and strains belonging to this species commonly show multi-drug resistance patterns [41]. Furthermore, both *P. multocida* and *M. haemolytica* are known to acquire and transfer antimicrobial resistance determinants through horizontal gene transfer [42], which is the main driver of intra- and inter-species antimicrobial resistance spread [43].

Interestingly, CCA and CCB inhibited the proliferation of *S. suis* at very low concentrations, equal to 3.75 mg L^−1^ and 7.50 mg L^−1^, respectively, whereas CP struggled to approach the MIC threshold. Considering that *S. suis* is a major bacterial pathogen in pigs, linked to systemic infections and meningitis and leading to increased mortality, reduced growth rates, and significant economic losses for pig producers, any products able to treat or limit its infection are of great interest for the pig industry [44]. Indeed, *S. suis* strains show resistance to multiple antimicrobials [44] and pose a zoonotic risk, especially to the workers exposed to infected animals [45]. Thus, these promising in vitro results suggest that the effectiveness of the chestnut compounds against *S. suis* should be adequately exploited in real-world scenarios.

Although all the compounds displayed comparable inhibition effect trends, hampering bacterial replication at the highest concentrations tested, none reached the MIC threshold for *S.* Typhimurium and *E. coli*. However, while the growth of *E. coli* was only slightly influenced by CCA and CCB, inhibition was observed when the *E. coli* strain was tested against CP. Indeed, exposure to increasing concentrations of CP was associated with a dose-dependent reduction in O.D._600_, with 15 mg L^−1^ of this compound resulting in a mean O.D._600_ increase equal to 0.057, which was just slightly above the adopted MIC threshold (i.e., an increase in turbidity of <0.05). Furthermore, significant differences in the mean O.D._600_ between CP and the other two compounds (vs. CCA mean of differences = −0.662, 95% CI −0.343/−0.982, *p* = 0.028 and vs. CCB mean of differences = −0.505, 95% CI −0.342/−0.668, *p* = 0.031) were observed. These findings indicate that CP could be a promising candidate as an alternative to the use of antimicrobials for the control and treatment of *E. coli* infections. *E. coli* is widespread in livestock production and known to carry multiple resistance genes [46]; therefore, any compound capable of inhibiting the growth of this bacterium could have a huge positive impact on livestock production.

Some of the polyphenol-mediated mechanisms of antibacterial activity are non-specific (e.g., oxidative stress), while other mechanisms only act on certain bacteria (e.g., toxin inhibition) [14,15]. In particular, Villanueva et al. [10] demonstrated an anti-biofilm activity of selected tannins inhibiting the growth of *S.* Typhimurium, *P. aeruginosa*, *E. coli*, and *S. aureus*. Since biofilm generation is a common trait of many bacteria, polyphenolic particles could be explored for their possible use as biofilm disruptors.

Plant phenolics have a potential anti-quorum sensing activity as well. Quorum sensing is the mechanism of bacterial cell-to-cell chemical communication, playing an important role in antibiotic resistance, biofilm formation, survival, proliferation, and toxin production; hence, the inhibition of this signaling process can contribute to the biological control of bacteria [47].

Several research groups [48,49] explored different types of nanoparticles for microbial inhibition, with outcomes suggesting a higher efficacy against Gram-negative bacteria compared to Gram-positive types. The responsible factor is thought to be the difference between the membrane composition of these two groups, as Gram-positive bacteria have a thick and exposed peptidoglycan layer. On the other hand, it was demonstrated that biocompatible composite nanofibers displayed good antibacterial performance against both Gram-negative *Escherichia coli* and Gram-positive *Staphylococcus aureus* [50].

Growth inhibition by phenolic-modified nanomaterials has been previously reported for tannic acid-modified nanoparticles, effectively inhibiting *Listeria monocytogenes* [51]. Diverse mechanisms can be employed by nanoparticles, including adhesion to microbial cells, generation of reactive oxygen species (ROS), and interference of bacterial growth kinetics; however, the specific action against bacteria requires a dedicated in-depth analysis [52,53].

The current findings underline the potential of CCB, CCA, and CP as antimicrobial agents against a range of bacterial pathogens. Furthermore, the results suggest that the antimicrobial activity of the colloidal materials is specific to certain bacterial species. Indeed, the variations in the observed effects on the different bacteria envisage the influence of pathogen-specific factors and chemical–physical characteristics on antimicrobial efficacy, pointing at the existence of more sophisticated growth-inhibitory paths. Further research is required to understand the correlation between physico-chemical properties and antimicrobial activity and study possible combinations of different colloids as actual alternatives to antibiotics. Moreover, although phenolic compounds are generally considered in terms of their beneficial properties, such as their antioxidant activity, their cytotoxicity is also an object of study in eukaryotic cells [54]. This prompts cytotoxicity assessments as crucial steps in promoting the use of colloidal polyphenols in real-world scenarios.

## 4. Materials and Methods

### 4.1. Materials

All chemicals, which were purchased with the highest purity available, were used without any additional treatment. NaOH was purchased from J.T. Baker (Avantor, Radnor, PA, USA). Ethanol 96% was purchased from VWR (Avantor, Radnor, PA, USA). 2,4,6-tripyridyl-2-triazine, chloride acid (HCl), ferric chloride (FeCl_3_), sodium acetate, sodium carbonate, gallic acid, ammonium ferrous sulfate, and ammonium ferrous sulfate were purchased from Merck (Darmstadt, Germany). All solutions were prepared using ultrapure water from a Genie Direct-Pure water device (RephiLe Bioscience Ltd., Shanghai, China), with a resistivity of at least 18.0 MΩ·cm. Blood Agar (BA) medium, Muller Hinton Agar (MHA), Brain Heart Infusion (BHI) broth, and tannic acid were obtained from Oxoid, Thermo Fisher Scientific (Waltham, MA, USA). All plasticware, including the plates for the minimum inhibitory concentration (MIC) measurements, was only used once.

The three phenolic compounds that are the object of the current study were gently provided by AINT s.r.l. (Advanced Iron Nano Technologies, Santa Croce, 510, Venice, Italy) as dried powders and in the form of water suspensions. AINT s.r.l. develops formulations for animal farming using selected materials from different large-scale worldwide manufacturers of wood by-products. Colloidal chestnut A and B (CCA and CCB) were extracted from chestnut trees in two distinct Italian regions, and colloidal pine (CP) was extracted from pine biomass.

### 4.2. Methods

Antioxidant activity determination was carried out using a UV-1800 spectrophotometer (Shimadzu, Columbia, MD, USA) according to the Folin–Ciocalteâu (FC) and ferric-reducing antioxidant power (FRAP) methods [55,56]. In the FC assay [56], gallic acid was used as the calibration standard. The FC assay was carried out by pipetting 1 mL of sample into a 10 mL test tube, followed by the addition of 1 mL of diluted FC reagent (i.e., 1 part of FC reagent and 2 parts of water). The mixture was vortexed (Vortex V1 plus, BOECO, Hamburg, Germany) and incubated for 3 min at room temperature. Then, 2 mL of 10% sodium carbonate solution was added, and the mixture was vortexed for 20–30 s (time 0). After 15 min at room temperature, the absorbance of the colored reaction product was measured at 750 nm. The total phenol (TP) content in the extracts was calculated from a standard calibration curve obtained with different concentrations of gallic acid, ranging from 0 to 37.5 mg L^−1^ (Figure S1). The results were first calculated as mg of gallic acid equivalent (GAE) per kg of dry weight and finally expressed as the normalized antioxidant effect.

The freshly prepared FRAP reagent contained 1 mmol L^−1^ of 2,4,6-tripyridyl-2-triazine and 2 mmol L^−1^ of ferric chloride (FeCl_3_) in sodium acetate 0.25 mol L^−1^ (pH = 3.6). A methanol-extracted volume of polyphenol sample (100 µL) was added to the FRAP reagent (1900 µL) and carefully mixed. After incubating the mixture at 20 °C for 4 min, sample absorbance was determined at 593 nm. Calibration was performed with a standard curve (0–1200 µg mL^−1^ ferrous ion), obtained via the addition of freshly prepared ammonium ferrous sulfate (Mohr’s salt, (NH_4_)_2_SO_4_·Fe(SO_4_)·6H_2_O). The FRAP values were calculated as µg mL^−1^ of ferrous ion (ferric-reducing power) from three determinations and finally expressed as the normalized antioxidant effect.

The zeta potential and size distribution of the colloidal materials were measured in water by dynamic light scattering (DLS) using a Zetasizer Nanoparticle analyzer ZEN3600 (Malvern Instrument, Malvern, UK). The LogNormal-function was used to obtain the statistical analysis on the size-distribution. High-resolution transmission electron microscopy (HR-TEM) micrographs of colloidal materials were attained with an HR-TEM TITAN 60–300 microscope equipped with an X-FEG-type emission gun (FEI, Thermo Fisher Scientific, Waltham, MA, USA). One drop of the as-obtained suspension was placed on a carbon-film-coated copper grid and dried at room temperature. In order to perform the stability tests, colloidal water suspensions were initially prepared at the final concentration of 1 g L^−1^ and then diluted to 50 mg L^−1^ under the different test conditions, including 96% ethanol, 3 M HCl, 3M NaOH, and pH values in 7–14 pH interval.

After one hour of incubation under stirring at room temperature, the materials were characterized through DLS and Uv-vis spectroscopy. Their antioxidant powder was assessed by the Folin–Ciocalteâu assay.

### 4.3. Minimum Inhibitory Concentration (MIC) Assay

Standardized inocula of six bacterial strains previously isolated from livestock (*E. coli*, *M. haemolytica*, *P. multocida*, *S. aureus*, *S.* Typhimurium, and *S. suis*) were used for the experiments. The list of the tested bacteria is reported in Table S1. Strains stored at −80 °C were resuscitated in duplicates in Blood Agar and incubated at 37 °C. After 24 h, a single colony from each plate was inoculated into 5 mL of Brain Heart Infusion (BHI) broth and incubated at 37 °C for 24 h under orbital agitation using a PSU-10i orbital shaker (Biosan, Riga, Latvia) within an IN75 incubator (Memmert, Schwabach, Germany). After this second incubation, the broth cultures were diluted to a final concentration of 5.0 Log10 CFU (colony-forming unit)/mL, which was used as the inoculum for the microtiter assays to assess the inhibitory effect of the phenolic compounds. The microtiter assays were carried out in triplicates over two weeks. Briefly, serial dilutions (15.00, 7.50, 3.75, 1.88, 0.94, and 0.47 mg L^−1^) of the phenolic compounds were prepared and loaded (50 µL) onto 96-well flat-bottom microtiter plates (Sarstedt, Nümbrecht, Germany). Each dilution was then inoculated with either 150 µL of standardized inoculum or 150 µL of BHI. Wells inoculated with BHI and the phenolic compounds were included to correct for potential absorbance interferences due to the phenolic compounds themselves. Negative control wells containing only the growth medium (without bacteria or nanoparticles) were included, as well as positive controls consisting of the inoculum without phenolic compounds. Before incubation (T0) at 37 °C for 24 (h), the optical density at 600 nm (O.D._600_) was measured using Spectrophotometer Multiskan GO Microplate Readers (Thermo Fisher Scientific, Waltham, MA, USA). Initial readings (T0) were taken to establish the baseline density. The O.D._600_ was then measured after 24 h of incubation. At both time-points, five measurements at five-minute intervals were carried out. The optical density (mean O.D._600_) of each phenolic compound dilution/inoculum combination was calculated as the average absorbance of the biological replicates over the two experiments. The standard deviation and coefficient of variation (CV) were also calculated to assess the data robustness (CV < 0.5). The minimum inhibitory concentration (MIC) was set as the lowest concentration of phenolic compounds that limited the increase in turbidity of all technical replicates to < 0.05, according to a published method [57]. The mean differences and the 95% confidence intervals (95% CI) were calculated, and significant differences were set at a *p* value < 0.05.

## 5. Conclusions

Antimicrobials are used in livestock production to cure diseased animals and prevent the dissemination of bacteria with zoonotic potential along the food chain. However, the overuse of antimicrobials in the food chain might contribute to the emergence of resistant bacteria, posing a threat to both animal and human health. The ability of the tested colloidal polyphenol materials to inhibit the growth of specific bacterial pathogens suggests their potential application in controlling and preventing infections in livestock. In particular, they displayed different antimicrobial activities against selected bacterial pathogens. The chestnut-derived colloids were effective against one of the most threatening antibiotic-resistant pathogens, i.e., *S. aureus*, but ineffective toward *E. coli*. On the other hand, the latter microorganism showed susceptibility to colloidal pine, a material which was, instead, ineffective against *S. aureus*. Indeed, if used in combination, such phenol nanoparticles may represent realistic alternatives to traditional antibiotics in livestock production.

The present interdisciplinary collaboration between biophysics, biotechnology, materials science, and microbiology paves the way for further research aimed at enhancing our comprehension of the principles determining the efficacy of these compounds, specifically with respect to their natural sources, antioxidant properties, and colloidal features. In addition, finding the correlation between their chemical–physical properties, mechanism of action, safety profile, in vivo efficacy, and, eventually, the in-field application conditions is necessary to optimize our understanding of these compounds and their broader implications in promoting sustainable and effective disease management in the livestock industry.

## Figures and Tables

**Table 1:** FTIR vibration attribution of the identified spectral feature wavelengths (cm^−1^) of CCA, CCB, CP, and TA, used as a control.

Spectral Identification	CCA (cm^−1^)	CCB (cm^−1^)	CP (cm^−1^)	TA (cm^−1^)
Stretching vibrations of the hydroxyl groups -OH	3300	3340	3360	3350
Symmetric stretching vibrations and antisymmetric -CH-	2930		2935 2850	
Carbonyl group stretching C=O	1725	1718		1701
Deformation vibrations of the aromatic backbone C-C	1605	1600 1512	1590 1510	1605 1534
Stretching vibrations of the aromatic backbone C-C	1445	1448	1456	1443
Stretching vibration of C-O	1283 1168	1305 1175	1265 1215 1130 1080	1308 1175 1080

**Table 2:** Differences in the mean O.D._600_ (AU/cm) among the tested compounds.

Bacteria	Compound	Mean O.D._600_ (AU/cm)	Comparator	Mean O.D._600_ (AU/cm)	Mean Difference	95% CI		*p* Value
						Lower	Upper	
*S. aureus*	CCA	0.228	CCB	0.155	0.074	0.005	0.143	0.031
	CP	0.322	CCA	0.228	0.094	0.040	0.147	0.041
	CP	0.322	CCB	0.155	0.167	0.106	0.229	0.014
*M. haemolitica*	CCA	0.255	CCB	0.214	0.040	−0.066	0.146	>0.05
	CP	0.223	CCA	0.255	−0.031	−0.181	0.118	>0.05
	CP	0.223	CCB	0.214	0.009	−0.178	0.195	>0.05
*P. multocida*	CCA	0.410	CCB	0.377	0.032	−0.070	0.135	>0.05
	CP	0.258	CCA	0.410	−0.151	−0.344	0.042	>0.05
	CP	0.258	CCB	0.377	−0.119	−0.350	0.112	>0.05
*S. suis*	CCA	0.120	CCB	0.229	−0.109	−0.222	0.004	>0.05
	CP	0.283	CCA	0.120	0.163	0.079	0.247	0.036
	CP	0.283	CCB	0.229	0.054	−0.117	0.224	>0.05
*S.* Typhimurium	CCA	0.593	CCB	0.656	−0.063	−0.203	0.076	>0.05
	CP	0.694	CCA	0.593	0.101	0.005	0.196	>0.05
	CP	0.694	CCB	0.656	0.037	−0.140	0.215	>0.05
*E. coli*	CCA	1.275	CCB	1.118	0.157	−0.024	0.339	>0.05
	CP	0.612	CCA	1.275	−0.662	−0.343	−0.982	0.028
	CP	0.612	CCB	1.118	−0.505	−0.342	−0.668	0.031

## Data Availability

The original data are available upon reasonable request to the corresponding author.

## Supplementary Materials

The following supporting information can be downloaded at https://www.mdpi.com/article/10.3390/ijms25179363/s1.
