# Supplementary material for: Highly Specific Polyphenolic Colloids as Alternatives to Antimicrobials in Livestock Production
Source: Int J Mol Sci. 2024 Aug 29;25(17):9363. doi: 10.3390/ijms25179363 (PMC11395071; doi:10.3390/ijms25179363)
Supplement: Supplementary file 1 [file ijms-25-09363-s001.zip › ijms-3161318-supplementary.pdf]

Supplementary Information

Corresponding author: Prof. Massimiliano Magro - Department of Comparative Biomedicine and Food Science, University of Padova, Agripolis, Viale dell'Università 16, 35020 Legnaro, Padua, Italy

Telephone: +39-049-8272916

Email: massimiliano.magro@unipd.it

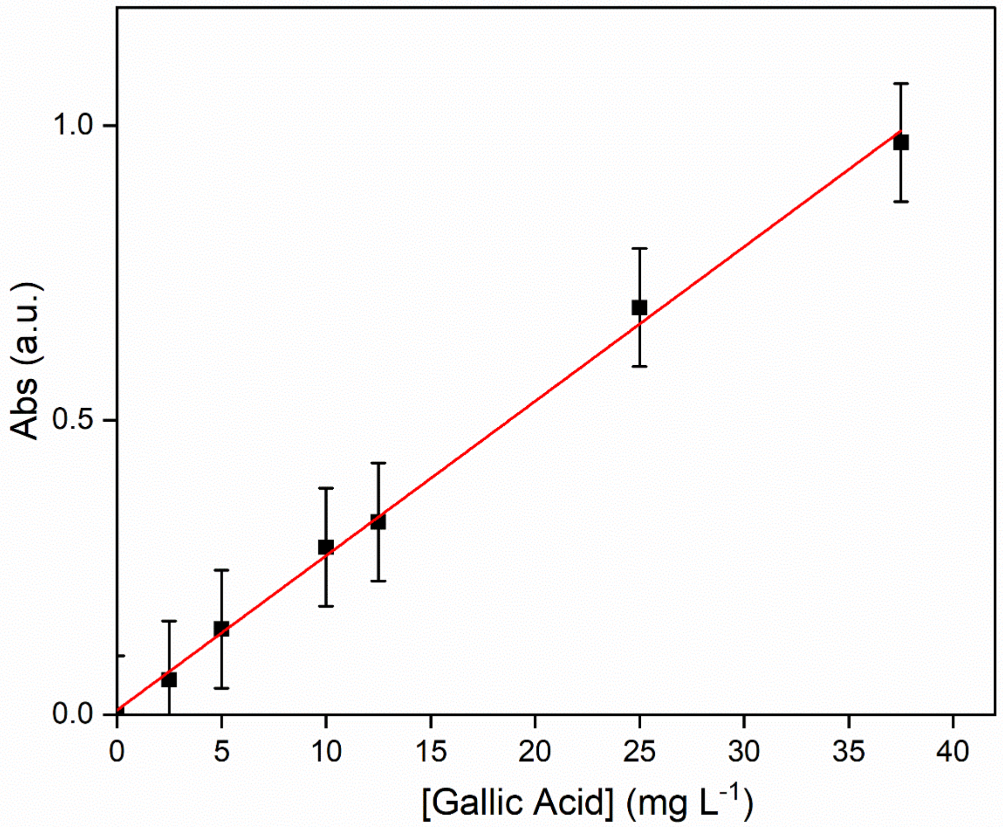

Figure S1. Folin–Ciocalteâu calibration curve built by plotting the Uv-vis absorbance of the assay

reaction product at 750 nm against increasing gallic acid, as a reference, in the concentration range from 0 to 37.5 mg L^{-1}.

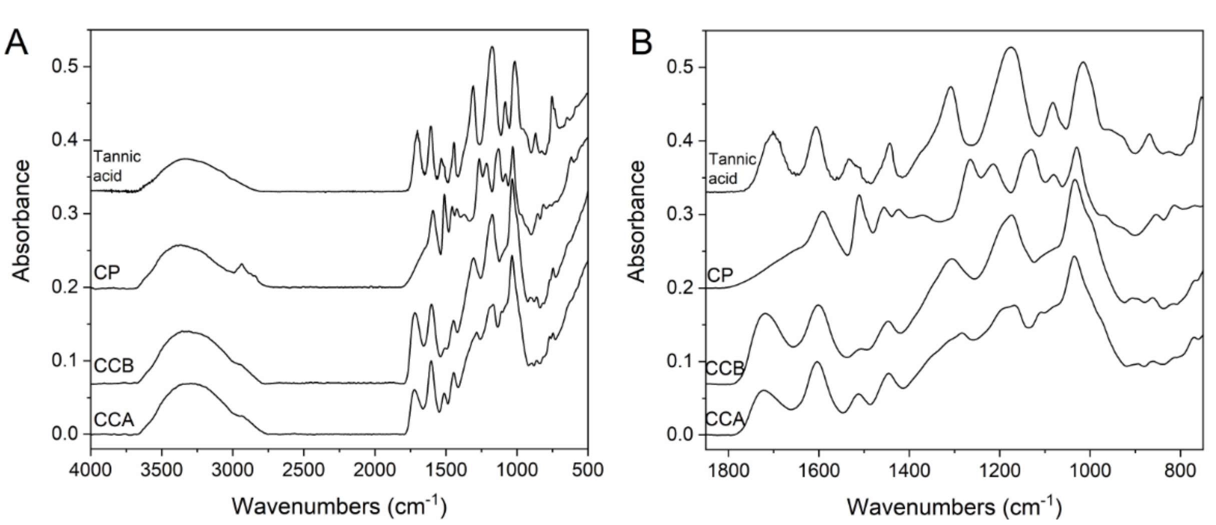

Figure S2. comparison of CCA, CCB, CP and commercial tannic acid FTIR profiles in (**A**) the 4000-400 cm^{-1} and (**B**) 1800-800 cm^{-1} wavelength intervals.

Host	Bacteria	Disease	Gram +/-	log cfu/ml
Bovine	*Pasteurella multocida*	Polmonitis	neg	7,94
	Staphylococcus aureus	Mastitis	pos	8,09
	Mannehimia haemolitica	Polmonitis	neg	7,59
Swine	*Streptococcus suis*	N.D.	pos	5,88
Chicken	Avian Pathogenic *Escherichia coli*	Colibacillosis	neg	11,06
	Salmonella Typhimurium	N.D.	neg	7,76

Table S1: list of bacterial strains included in the study.
